# You Pretend, I Laugh: Associations Between Dyadic Pretend Play and Children's Display of Positive Emotions

**DOI:** 10.3389/fpsyg.2021.669767

**Published:** 2021-06-23

**Authors:** Zhen Rao, Jenny L. Gibson

**Affiliations:** Centre for Research on Play in Education, Development and Learning, Faculty of Education, University of Cambridge, Cambridge, United Kingdom

**Keywords:** pretend play, positive emotions, pretend themes, peers, school-aged children, dyadic analysis

## Abstract

**Background:** Understanding how pretend play is related to positive emotions is important for supporting children's development and promoting their wellbeing. However, previous studies have mainly examined this association at individual levels and overlooked the potential links at interpersonal levels. This is an important knowledge gap because pretend play is commonly performed in social contexts. The current study investigates how peer pretend play is associated with children's display of positive emotions at both individual and dyadic levels.

**Methods:** One hundred and eight Chinese children (*M*_age_ = 8.95 years, *SD* = 0.99, 51.9% girls) were observed playing in peer dyads with toys. An interaction of 10 min was coded for each child's pretend play behavior, social and emotional pretend play themes, and display of positive emotions. Multilevel modeling was used to examine age and gender differences in peer pretend play. Actor–Partner Interdependence Models (APIM) were estimated to test the hypothesized associations between dyadic pretend play and children' display of positive emotions.

**Results:** Compared to children whose playmates engaged in less pretend play, children whose playmates engaged in more pretend play were more likely to display positive emotions (*p* = 0.021). Additionally, children's display of positive emotions was predicted by both their own (*p* = 0.027) and their playmate's (*p* = 0.01) pretend play with emotional themes. Compared to younger children, older children were less likely to engage in pretend play (*p* = 0.002), but more likely to engage in pretend play with social themes (*p* = 0.03) when the total frequency of pretend play was controlled for. Boys were 4.9 times and 2.16 times as likely as girls to create aggressive pretend themes (*p* < 0.001) and non-aggressive negative pretend themes (*p* = 0.007), respectively. No significant gender differences were found in positive pretend themes.

**Conclusions:** Pretending with peers may increase not only children's own, but also their play partner's display of positive emotions. Pretend play may not simply decline in middle childhood as previously assumed.

## Introduction

Pretend play has been widely studied as a window to child development. One important research agenda that has received growing attention is to understand how pretend play is related to children's emotions (e.g., Fiorelli and Russ, 2012; Lindsey and Colwell, 2013; Rao and Gibson, 2019). Also receiving increasing recognition in the field is the need to examine the complexity of social pretend play. This includes measuring genuine pretend play in social contexts, accounting for the interdependence of social play behavior, and adopting a multilevel perspective (e.g., both individual and interpersonal perspectives; Bergen, 2013; Lillard et al., 2013; Weisberg et al., 2013; Gibson et al., 2019). A further movement in the research field is to understand pretend play beyond early childhood and beyond Western cultures, with investigators starting to examine pretend play in school-aged children (e.g., Smith and Lillard, 2012; Howard et al., 2017) and in non-Western cultures (e.g., Roopnarine, 2011; Gaskins, 2013). In line with these important research agendas, our study takes a dyadic approach to investigate dyadic pretend play in school-aged Chinese children and examine its association with children's display of positive emotions at both individual and dyadic levels.

### Theoretical Background

Pretend play has been conceptualized as play activities that include at least one of the three fundamental forms of pretense: object substitution, pretend attribution of properties, and imaginary objects (Leslie, 1987). As a form of play activity, pretend play also has essential characteristics of play, such as spontaneity, autonomy, being performed for its own sake and intrinsically rewarded (Smith, 1986). Typically emerging during the second year of life, pretend play evolves with children's cognitive, social, emotional, and cultural development, and is commonly performed in social contexts. The capacity for meta-representation and the ability to understand mental states have been proposed as underlying children's ability to engage in pretend play and understand other's pretense (Leslie, 1987). Social interaction with siblings and peers, available play venues and materials may also affect the frequency and content of children's pretend play (Rubin et al., 1983). Under the influence of possible biological propensity and gendered socialization, children may adopt play behaviors that are consistent with their gender roles (Martin and Halverson, 1981). Parental beliefs of the value of pretend play and cultural attitudes toward it could also affect the frequency and content of children's pretend play (Gaskins, 2013).

While a developmental trend of increasing frequency and complexity of pretend play during early years is widely agreed, there is no consensus on the developmental trend of this phenomenon in later childhood. Although Piaget (1962) suggested a decline in pretend play between 8 and 12 years, Harris (2000, p. 28) regarded pretend play as an indication of “a lifelong mental capacity to consider alternatives to reality.”

Performed in a non-literal way, pretend play has been proposed as a way of minimizing the consequences of trying out emotional experiences in real life situations, therefore providing a safer context for children to explore their emotions (Erikson, 1995; Lillard, 2017). Pretend play has dual affective effects, an example of which is a child “weeps in play as a patient, but revels as a player” (Vygotsky, 1933, p. 549). Providing children with a sense of control and autonomy, pretend play may serve the function of self-healing (Erikson, 1995). When enacted socially, pretend play creates a psychological “frame” and provides play partners with the messages “It's just play,” “Don't take it seriously” (Bateson, 1972). Similar to adolescent's self-disclosure, pretend play may provide a safe mechanism for younger children to express their fears and worries with friends (Gottman, 1986). The incongruous condition between the real object and the pretend actions directed toward it also provides the context for children to experience humor and positive emotions (McGhee, 1979).

### Pretend Play in School-Aged Children

Only a limited number of studies have investigated pretend play in school-aged children. A focus group study in the UK found that pretend play was reported by 7- to 11-year-olds as a common play activity, contrary to the notion of pretense being relevant only in early childhood (Howard et al., 2017). Using self and parent report, Taylor et al. (2004) found that imaginary companions were as common among 6- and 7-year-olds as they were among 3- and 4-year-olds. A retrospective questionnaire study with 113 undergraduates in the US found that the average age at which the respondents ceased to engage in pretend play was 11 years (Smith and Lillard, 2012). Compared to their female counterparts, male respondents reported themselves to cease pretend play at older ages.

The existing evidence, however, has mainly come from studies using self- and other-report and with children in Western cultures. Little is known about how school-aged children in non-Western cultures engage in pretend play. Although a recent study reported that pretend play was a popular type of social play among 8- to 11-year-old Chinese children (Rao et al., 2020), it was based on small-scale interview rather than direct observation. Given the potential bias from self- and other-report, new evidence is needed which directly observes pretend play in middle childhood, and especially in social contexts. As play behavior can be influenced by cultural factors such as parental beliefs on the value of play, it is important to examine pretend play in different cultures to understand the similarities and variations in middle childhood pretend play (Roopnarine, 2011; Gaskins, 2013).

### Links Between Pretend Play and Emotional Expression

Studies on how pretend play is associated with emotional expression have been largely conducted with young children. Lindsey and Colwell (2013) observed 122 preschoolers' naturally occurring play behavior with peers in a child care setting at two time points (1 year apart). At Time 1, children's engagement in sociodramatic play (e.g., complex social role play) was positively related to their positive emotional expressiveness and negatively related to their negative emotional expressiveness. At Time 2, boys (but not girls) who had engaged in more sociodramatic play at Time 1 were found to express more positive emotion. Dunn and Hughes (2001) found that 4-year-olds who displayed more negative emotions than a control group with typical emotional regulation, enacted a higher proportion of violent pretend play, and a lower proportion of magic pretend play. Youngblade and Dunn (1995) observed fifty 33-month-old children at home with their siblings and mothers. They found that children who created a greater diversity of pretend themes were more likely to express affection to their mothers.

Observing pretend play in social contexts, the above studies suggest close relations between pretend play and emotional expression in early childhood in Western cultures. Whether these relations remain in middle childhood and in other cultures, however, cannot be assumed and need to be answered by empirical studies. A further limit of the existing evidence is its use of an individual lens, with potentially important associations at interpersonal levels being overlooked.

### The Current Study

The current study aims to address two questions. First, it investigates whether there are age and gender differences in the frequencies and themes of pretend play observed in 7- to 10-year-old Chinese peer dyads. Second, it investigates whether there are individual and dyadic associations between pretend play and children's positive emotions.

As discussed above, a lack of evidence on pretend play in middle childhood and in non-Western cultures has hindered our understanding of the developmental trajectory and cultural variations of pretend play. This is critical not just for understanding play for its own sake, but also for understanding the pathways through which play may affect other aspects of development and the extent to which these are universal processes of child development, observable in all cultures. For instance, compared to Euro-American parents, Asian parents were found to place less value on play and more importance on academic achievement (Parmar et al., 2004). This may affect children's opportunity to engage in pretend play, which could have implications for their social and emotional development. Compared to Irish American children, Chinese children were found to engage in less pretend play related to fantasy and caretaking, and more pretend play related to routine social interactions with non-kin adults (Haight et al., 1999). Furthermore, recent evidence has suggested that both children's social pretend play and emotional expression can be significantly influenced by their social partners (Gibson et al., 2019; Lunkenheimer et al., 2020). By taking a dyadic lens, our study allows the examination of interpersonal associations between pretend play and emotional expression that have been previously overlooked.

## Methods

### Participants

One hundred and thirty-six children (age range: 7–10 years, *M*_age_ = 9 years, *SD* = 0.97, 52.2% female) from a boarding school in Guangzhou, China were invited to participate in the study. Seventy-two children were from year two (*M*_age_ = 8 years and 2 months, *SD* = 0.33, 45.8% female) and 64 children were from year four (*M*_age_ = 9 years and 11 months, *SD* = 0.32, 59.4% female). All participants were Chinese and spoke Mandarin. Following the review and approval of the study by the institution's ethics committee, letters were circulated to the school to recruit participants. Informed written parental consent and verbal child assent were obtained for all participants prior to data collection.

The 136 children were paired into 68 peer dyads according to their nomination of peers with whom they would like to take part in the study. Among the 68 dyads, eight dyads were excluded for analysis due to inadequate recording quality. A further five dyads were excluded because neither of the children in the dyads had nominated the other as playmate. Of the remaining 55 dyads, only one was mixed-gendered and was excluded from the analysis for the purpose of using gender as a between-group variable. This resulted in a final sample of 54 same-sex dyads (108 children, *M*_age_ = 8 years and 11 months, *SD* = 0.99, 51.9% female), which included 32 mutually nominated and 22 one-way nominated dyads. The mutually nominated and one-way nominated dyads did not differ significantly in total pretend [*t*_(106)_ = 1.19, *p* = 0.237], social pretend themes [*t*_(106)_ = 1.08, *p* = 0.281], emotional pretend themes [*t*_(106)_ = 1.18, *p* = 0.241], or displayed positive emotion [*t*_(106)_ = 0.85, *p* = 0.397].

### Procedure

Dyads were invited to play with toys in play areas set up in a school art room for 20 min, with each pair being video recorded separately during play. No request, example, or cue for pretend play or other type of play were given to the children. The researcher left the children to play on their own and stayed in a corner of the room reading paperwork.

### Measure

#### Play Materials

Each pair of children was provided with a toy set consisting of the same materials (e.g., Lego figures, bricks, and non-Lego mini bear figures, see Supplementary File for photos of all the play materials provided). The play materials were selected with the aim to elicit pretend play while minimizing the potential influence of toys on the pretend content (i.e., neutral human figures and bricks were used rather than those associated with specific themes). A pilot study showed that these toys were fit for purpose.

#### Scoring Method

The play videos were transcribed and analyzed using the software ELAN Version 5.1 (ELAN, 2017). Based on previous research (e.g., Fein, 1989; Dunn and Hughes, 2001; Russ, 2004) and the analysis of 10 randomly selected videos, a coding scheme was developed to analyze the videos in two steps (Rao, 2019).

##### Step One

Each child's verbal and non-verbal behavior was coded at 5-s intervals for 10 min, a duration consistent with previous research (e.g., Fehr and Russ, 2013; Gibson et al., 2019). For each child, 120 5-s segments were analyzed in three aspects in this step (see Appendix 1 in Supplementary Material for descriptions and examples of codes). The first aspect “Displayed emotions” examined emotional expression observed for each child. For each 5-s interval, a child was assigned one of the two mutually exclusive codes: “Displayed positive emotions” (e.g., smiling, laughing) and “Displayed neutral emotions.” A code “Displayed negative emotions” was initially included but was dropped due to its rare occurrence. The second aspect “Speech and social interaction” examined how each child spoke and interacted their play partner. For each 5-s interval, a child was assigned one of the three mutually exclusive codes: “Speaking” (e.g., talking to the play partner), “Not speaking—attentive” (e.g., looking at the play partner without speaking), and “Not speaking—solitary” (e.g., focusing on building bricks without speaking or looking at the play partner). The third aspect “Play type” examined whether a child engaged in pretend play at each 5-s interval and included two mutually exclusive codes: “Pretend play” and “Not pretend.” “Pretend play” was coded when a child enacted a pretend scenario (e.g., putting toy near mouth as if eating) or explicitly spoke about (e.g., planning, labeling, negotiating, or clarifying) a pretend role or behavior (e.g., “Can I be the Mum?”).

##### Step Two

Segments that had been assigned the code “Pretend play” for the “Play type” aspect in step one were examined at step two for two aspects: “Social pretend content” and “Emotional pretend content.” “Social pretend content” consisted of two mutually exclusive codes: “Social pretend themes” (e.g., “They are getting married”) and “Non-social pretend themes” (e.g., “This is the light”). “Emotional pretend content” consisted of seven codes: “Positive pretend emotions” (e.g., “The cat is happy”), “Positive pretend scenarios” (e.g., “They are warm and comfortable now”), “Pretend superpower” (e.g., “This restaurant can fly”), “Negative pretend emotions” (e.g., “He is angry”), “Aggressive pretend scenarios” (e.g., “They are fighting”), “Non-aggressive negative pretend scenarios” (e.g., “The dog is ill”), and “Neutral pretend scenarios” (e.g., “They are swimming”). The first six codes were not mutually exclusive and were coded when relevant theme was observed in each five second segment. Segments that did not include themes in any of the first six codes were assigned the code “Pretend neutral scenarios” (see Appendix 2 in Supplementary Material for descriptions and examples of codes).

##### Reliability Coding

A random selection of videos of 22% (*N* = 26) of the 120 children whose videos had adequate quality were individually coded by the first author and an independent coder unaware of the hypotheses of the study. Cohen's kappa for inter-rater reliability ranged from 0.81 to 0.9 (*p*s < 0.001) for the first step, and from 0.79 to 0.89 (*p*s < 0.001) for the second step, indicating good inter-rater reliability (Cohen et al., 2011).

### Data Preparation

Nine variables were derived from the play observations (Table 1). Due to low occurrences of the codes “Positive pretend emotions” and “Pretend superpower,” these were combined with the code “Positive pretend scenarios” to create the variable “positive pretend themes.” Due to low occurrences of the code “Negative pretend emotions,” this was combined with “Non-aggressive negative pretend scenarios” to create the variable “non-aggressive negative pretend themes.” The variable “emotional pretend themes” summed up the scores of “positive pretend themes,” “non-aggressive pretend negative themes,” and “aggressive pretend themes.”

**Table 1:** Descriptive statistics and intra-class correlations (ICC) for play observation variables (*N* = 108).

	**Total pretend**	**Speech**	**Displayed positive emotion**	**Social interaction**	**Social pretend themes**	**Emotional pretend themes**	**Positive pretend themes**	**Non-aggressive negative pretend themes**	**Aggressive pretend themes**
Mean	22.42	70.58	33.73	95.77	11.17	5.95	2.26	1.69	2.00
*SD*	13.38	18.36	19.26	17.19	8.80	5.86	2.50	2.56	3.28
Range	1–57	26–107	6–82	54–120	0–36	0–25	0–13	0–14	0–13
Skewness (*SE*)	0.59 (0.23)	−0.12 (0.23)	0.67 (0.23)	−0.48 (0.23)	0.69 (0.23)	1.05 (0.23)	1.54 (0.23)	2.44 (0.23)	1.79 (0.23)
Kurtosis (*SE*)	−0.09 (0.46)	−0.58 (0.46)	−0.37 (0.46)	−0.69 (0.46)	−0.22 (0.46)	0.33 (0.46)	2.91 (0.46)	7.63 (0.46)	2.34 (0.46)
ICC	0.41	−0.04	0.47	0.65	0.35	0.86	0.56	0.66	0.77

*The scores for each variable equal to the numbers of 5-s segments where a child was observed showing the behavior out of 120 5-s segments coded*.

As children's utterances during play may affect their frequency of pretend utterances, the variable “speech” was created as a control variable which scored the number of segments when a child spoke (coded as “Speaking”). As children's interactions with their playmates may affect their display of positive emotions, the variable “social interaction” was created as a control variable which scored the number of segments when a child interacted with playmate (coded as “Speaking” or “Not Speaking—Attentive”).

### Analytic Plan

Because children were observed in dyads and their behavior may be affected by each other, and because the dyads in this study were indistinguishable (i.e., cannot be distinguished in a meaningful way), intra-class correlation (ICC) were calculated for all observational variables to measure the non-independence of the dyadic data (Kenny et al., 2006). Intra-class correlation measures the extent to which the variables of individuals in the same dyad resemble each other as compared to those from individuals in different dyads (Rasbash et al., 2017). If ICCs indicated the non-independence of the data, multilevel modeling would be used to account for this interdependence. Two-level random intercept models using restricted generalized least squared (RGLS) would be run in MLwiN version 3.02 (Charlton et al., 2017) to test the following hypotheses.

#### Main Hypothesis 1

It was hypothesized that there were age and gender differences in the frequencies and themes of the dyadic pretend play. This hypothesis was not directional, because little evidence has been reported on these differences in middle childhood. This hypothesis was addressed by testing three sub-hypotheses. Firstly, it was hypothesized that age and gender predicted each child's score on total pretend, controlling for their score on speech. Secondly, it was hypothesized that age and gender predicted each child's score on social pretend themes, controlling for their score on total pretend. Thirdly, it was hypothesized that age and gender predicted each child's scores on emotional pretend themes, controlling for their score on total pretend.

Four two-level models (individual at level 1 and dyad at level 2) were compared to test each sub-hypothesis. The first model was an intercept only model with no explanatory variable. The second model added age and speech (for the first sub-hypothesis) or added age and total pretend (for the second and third sub-hypotheses) as level-1 predictors to the intercept only model. The third model added gender as a level-2 predictor to the second model. The fourth model added the interaction between age and gender to the third model.

#### Main Hypothesis 2

It was hypothesized that children's display of positive emotions was predicted by their frequencies and emotional themes of pretend play at both individual and dyadic levels. This hypothesis was addressed by testing two sub-hypotheses using the Actor–Partner Interdependence Model (APIM) (Kenny et al., 2006). Firstly, it was hypothesized that each child's score on displayed positive emotion was positively predicted by their own score and their playmate's score on total pretend, controlling for the child's own score and their playmate's score on social interaction. Secondly, it was hypothesized that each child's score on displayed positive emotion was positively predicted by the child's own score and their playmate's score on emotional pretend themes, controlling for the child's own score and their playmate's score on social interaction.

## Results

### Descriptive Statistics

As can be seen from Table 1, children on average engaged in pretend play in 18% of the 120 5-s segments (10 min) and displayed positive emotions in 28% of these segments. Intra-class correlations for eight out of the nine variables (except for speech) varied from 0.35 to 0.86, indicating that 35–86% of the total residual variations of these variables were due to differences between dyads. The magnitude of ICCs warranted the use of multilevel modeling.

#### Main Hypothesis 1: Age and Gender Differences in Pretend Play

*Sub-hypothesis 1.1: Age and gender predict each child's score on total pretend, controlling for their score on speech*.

Model comparison indicated that the model using age and speech to predict total pretend had the best fit. This model indicated that children's score on total pretend is significantly predicted by age (b = −3.9, *p* = 0.002) and speech (b = 0.45, *p* < 0.001) (Table 2). Controlling for speech, children's engagement in pretend play decreased for an average of 3.9 units (each unit is a 5-s segment) for each 1-year increase in age.

**Table 2:** Multilevel model parameter estimates for age and gender differences in three pretend variables (*N* = 54 dyads).

	**Response**					
	**Total pretend**^****a****^		**Social pretend themes**^****a****^		**Emotional pretend themes**^****b****^	
**Predictor**	***B***	***SE***	***B***	***SE***	***B***	***SE***
Age	−3.90^*^	1.26	1.10^*^	0.51		
Speech	0.45^*^	0.04				
Total pretend			0.56^*^	0.03	0.05^*^	0.01
Gender					0.43^*^	0.19

*B, parameter estimate; SE, standard error of the estimate. All continuous predictors are grand mean centered. The reference category for gender is female*.

* *p < 0.05*.

a *Models were built with one normally distributed response variable*.

b *Model was built with one negative binomial response variable models because the variable was severely positively skewed*.

*Sub-hypothesis 1.2: Age and gender predict each child's score on social pretend themes, controlling for their scores on total pretend*.

Model comparison indicated that the model using age and total pretend to predict social pretend themes had the best fit. This model indicates that children's score on social pretend themes is significantly predicted by age (b = 1.1, *p* = 0.03) and total pretend (b = 0.56, *p* < 0.001) (Table 2). Controlling for total pretend, children's score on pretend social themes increases by an average of 1.1 units (each unit is a 5-s segment) for each 1-year increase in age.

*Sub-hypothesis 1.3: Age and gender predict each child's score on emotional pretend themes, controlling for their scores on total pretend*.

Model comparison indicated that a model using gender and total pretend to predict emotional pretend themes showed the best fit. This model indicated that children's score on emotional pretend themes was significantly predicted by gender (b = 0.43, *p* = 0.002) and total pretend (b = 0.05, *p* < 0.001) (Table 2). Specifically, boys were 1.54 times as likely as girls to create emotional pretend themes.

As pretend emotional themes consisted of three sub-themes (i.e., positive, aggressive, and non-aggressive negative themes), additional models were run to examine which sub-themes boys and girls engaged in differently. The results indicated that boys were 4.9 times and 2.16 times as likely as girls to create aggressive pretend themes (*p* < 0.001) and non-aggressive negative pretend themes (*p* = 0.007), respectively. No significant gender differences were found in positive pretend themes.

#### Main Hypothesis 2: Relations Between Display of Positive Emotions and Pretend Play

*Sub-hypothesis 2.1: Each child's score on displayed positive emotions was positively predicted by their own score and their partner's score on total pretend, controlling for the child's own score and their partner's score on social interaction*.

The estimates of the APIM model indicated that a child's score on displayed positive emotions was significantly predicted by their own score on social interaction (b = 0.52, *p* < 0.001) and their partner's score on total pretend (b = 0.33, *p* = 0.021) (Table 3). Controlling for social interaction, a child was more likely to display positive emotions when their partner engaged in more frequent pretend play. This model explained 17% of variance at child level and 17% of variance at dyad level.

**Table 3:** Parameter estimates for two actor-partner interdependence models (*N* = 54 dyads).

		**Sub-hypothesis 2.1**			**Sub-hypothesis 2.2**		
		**Displayed positive emotions**			**Displayed positive emotions**		
	**Predictor**	***B***	***SE***	***p***	***B***	***SE***	***p***
Actor	Social interaction	0.52^*^	0.13	0.000	0.48^*^	0.11	0.000
	Total pretend	0.08	0.14	0.579			
	Emotional pretend themes				0.64^*^	0.29	0.027
Partner	Social interaction	−0.22	0.19	0.261	−0.16	0.11	0.158
	Total pretend	0.33^*^	0.14	0.021			
	Emotional pretend themes				0.93^*^	0.29	0.01

*B, parameter estimate; SE, standard error of the estimate. All continuous predictors are grand mean centered*.

* *p < 0.05*.

*Sub-hypothesis 2.2: Each child's score on displayed positive emotion was positively predicted by the child's own score and their play partner's score on emotional pretend themes, controlling for the child's own score and their partner's score on social interaction*.

The estimates of the APIM model indicated that a child's score on displayed positive emotion was significantly predicted by their own score on social interaction (b = 0.48, *p* < 0.001), their own score on emotional pretend themes (b = 0.64, *p* = 0.027) and their partner's score on emotional pretend themes (b = 0.93, *p* = 0.01) (Table 3). Controlling for social interaction, a child was more likely to display positive emotions when they or their partner engaged in more frequent pretend play with emotional themes. This model explained 15% of variance at child level and 40% of variance at dyad level.

## Discussion

The present study takes a dyadic approach to examining pretend play in 7- to 10-year-old Chinese children and its relations to their display of positive emotions. An APIM revealed that children were more likely to display positive emotions when their play partner engaged in more frequent pretend play, whereas children's own pretend play frequency did not predict their own display of positive emotions. Additionally, children were more likely to display positive emotions when they or their play partner engaged in more pretend play with emotional themes. Although previous studies have documented positive relations between pretend play and positive emotions (e.g., Hoffmann and Russ, 2012; Lindsey and Colwell, 2013), none of them has examined how a partner's pretend play is related to a child's positive emotions. Significant partner effects found in the current study suggest that a child's expression of positive emotions may be promoted by their social partner's pretend behavior. Acting in a non-literal way may send positive emotional messages (e.g., “it is safe,” “it is fun”) to social partners, who can be encouraged to express positive emotions. As these findings are correlational, an alternative explanation is that children are encouraged to pretend when social partners show positive emotions. An important area for future research is to investigate whether encouraging social pretend play would increase children's expression of positive emotions, which has implications for children's emotional wellbeing.

The current study found significant age and gender differences in the frequencies and themes of pretend play in school-aged children. When children's speech was adjusted for, younger children pretended more frequently than did older children. Such an age difference may seem consistent with Piaget's (1962) claim that pretend play declines after the age of seven and eight. However, we also found an increase of social pretend themes with age, and no significant age difference in the emotional pretend themes. This suggests that older children may spend less time engaging in pretend play, but when they do, their pretense tends to be social in nature. A further possible explanation is that older children in China are under more pressure on academic performance and less encouraged to play by parents and teachers compared to younger children (Parmar et al., 2004). Although the overall incidence of pretend emotional themes was low, the current study found that boys were more likely to pretend with aggressive and non-aggressive negative themes. This is consistent with evidence from Western contexts and in early childhood (Dunn and Hughes, 2001; Russ, 2004; Marcelo and Yates, 2014). Such gender discrepancies may be due to a combination of biological, socialization, and contextual factors (Chaplin, 2015). For example, children may adopt play behaviors that are consistent with their gender roles (Martin and Halverson, 1981). The context of playing with same-sex close friends in the current study may also have encouraged boys' engagement in pretend play with aggressive and non-aggressive negative themes (Martin and Fabes, 2001).

Different from studies which request children to pretend (e.g., asking children to make up or continue a story), the present study measured pretend play spontaneously initiated and engaged in by children with familiar peers. This is important for the validity of the measure, as spontaneity and autonomy have been argued to be essential characteristics of play (Bergen, 2013). Nevertheless, this study is limited due to its use of an opportunity sample, its cross-sectional design and that not all dyads in the study were mutually nominated friends, which constrains its generalizability. Future studies are warranted to use more diverse samples, longitudinal designs, and to include different friendship types.

## Conclusions

The current study takes a dyadic approach and reveals the associations between peer pretend play and children's display of positive emotions at both individual and interpersonal levels. Compared to children whose playmates engaged in less pretend play, children whose playmates engaged in more pretend play were more likely to display positive emotions. In addition, pretending with emotional themes was found to increase the chance of a child, as well as their playmate, showing positive emotions. These findings provide important interpersonal perspectives that have been overlooked in previous research and highlight the social nature of pretend play and emotional expression.

A further contribution of our study is to provide new evidence on the age and gender differences in the frequencies and themes of pretend play among school-aged children in a Chinese context. Our study suggests a decrease of pretend play between the ages of 7 and 10 years of age. However, when controlling for total frequency of pretend play, we found an increase of social pretend themes with age and no significant age difference in emotional pretend themes. These findings call for further investigation of the developmental trajectory of pretend play and its developmental affordance in middle childhood.

## Data Availability Statement

The raw data supporting the conclusions of this article will be made available by the authors, without undue reservation.

## Ethics Statement

The studies involving human participants were reviewed and approved by Research Ethics Committee, Faculty of Education, University of Cambridge. Written informed consent to participate in this study was provided by the participants' legal guardian/next of kin.

## Author Contributions

ZR and JG both contributed to the conceptualization and methodology of the study, the interpretation of the analysis, writing and revision of this manuscript, and approving the final version to be published. ZR conducted the data collection and the data analysis. JG supervised the study.

## Conflict of Interest

The authors declare that the research was conducted in the absence of any commercial or financial relationships that could be construed as a potential conflict of interest.
