# Supplementary material for: You Pretend, I Laugh: Associations Between Dyadic Pretend Play and Children's Display of Positive Emotions
Source: Front Psychol. 2021 Jun 23;12:669767. doi: 10.3389/fpsyg.2021.669767 (PMC8262317; doi:10.3389/fpsyg.2021.669767)
Supplement: Supplementary file 1 [file Table_1.docx]

**Appendix 1**

*Three Aspects Coded in the First Step of Video Analysis*

| Aspect | Code | Description | Example |
| --- | --- | --- | --- |
| Displayed  Emotions | Displayed Positive Emotions | Showing obvious facial expression of positive emotions | Smiling, laughing, cheek raising, mouth opening |
|  | Displayed Neutral Emotions | Not showing obvious facial expression of positive or negative emotions | Facial change that is not obvious |
| Speech  &  Social Interaction | Speaking | Child speaks | “What should we play?” |
|  | No Speaking  - Attentive | Not speaking, but giving attention to playmate or what the playmate is doing | Child looks at playmate’s toy without speaking |
|  | No Speaking  - Solitary | Not speaking and not giving attention to playmate or playmate’s behaviour | Child focuses on building bricks without speaking or looking at playmate |
| Play Type | Pretend Play | Acting out pretend scenarios, including:  Speaking or making sound as a pretend character  Moving a toy or one’s own body to imitate actions (e.g., dancing, fighting) of a pretend character  Speaking to a toy as if is a pretend character, or explicitly speaking about a pretend scenario, including planning, labelling, negotiating, commenting, or asking for clarification | “I’m a superhero.”  “Miao!”  “Let’s make a zoo.”  “This is a baby.”  “Can I be the Mum?”  “Why is the cat sleeping there?” |
|  | Not pretend | Behaviour that does not indicate pretence | “This block does not fit in here.” |

*Note.* The 10 minutes of coding started from one minute after the pair was left to play on their own and excluded the segments where a child’s face could not be seen (e.g., out of camera).

**Appendix 2**

*Two Aspects Coded in the Second Step of Video Analysis*

| Aspect | Code | Description | Example |
| --- | --- | --- | --- |
| Pretend Social  Content | Pretend Social Themes | Includes any one of the following:  1) pretend social character, e.g., family members, friends, enemy, doctor, teacher  2) pretend behaviour involving more than one person or taking place in a social context, e.g., going to school, shopping, cooking, fighting  3) mention social rules in pretend scenario | “He is seeing the doctor.”  “They’re getting married.”  “You shouldn’t let others see you doing a wee.” |
|  | Pretend Non-Social Themes | Pretend scenarios that do not fit into above categories | “This is the light.”  “Here is a bowl.” |
| Pretend Emotional  Content | Pretend Positive Emotions | Directly mentions positive emotions in pretend scenarios | “The bear is very happy.” |
|  | Pretend Positive Scenarios | Includes any one of the following:  1) evaluate the pretend scenarios in positive ways  2) related to fulfilment of the need for connectedness, e.g., attachment, friendship, bonding, prosocial behaviour  3) related to fulfilment for the need for health and safety, e.g., caregiving, helping, protecting, curing, guarding, recovery from illness  4) related to self-fulfilment, feeling of accomplishment, e.g., play, winning | “He says, this is yummy.”  “They are warm and comfortable now.”  “The doctor cured him.”  “He’s guarding the palace.”  “They’re playing.” |
|  | Pretend Super-power | Pretend superpower, e.g., magic, flying, superpower | “This restaurant can fly.”  “She is invisible.” |
|  | Pretend Negative Emotions | Directly mentions negative emotions in pretend scenarios | “He is bored.”  “He’s angry.” |
|  | Pretend Aggressive Scenarios | Related to aggression, e.g., fighting, punishing, kicking, killing | “They are at war.” |

**Appendix 2 (continued)**

| Aspect | Code | Description | Example |
| --- | --- | --- | --- |
| Pretend Emotional  Content | Pretend Non-Aggressive Negative Scenarios | Includes any one of the following but does not include aggressive scenarios:  1) evaluate the pretend scenarios in negative ways  2) opposite to the fulfilment of the need for connectedness, e.g., ignorance, rejection, isolation, abandon, cheating, separation, not obeying social rules  3) opposite to the fulfilment for the need for health and safety, e.g., illness, unhelpfulness, danger  4) opposite to self-fulfilment, related to helplessness, ineptness, failure, e.g., losing competition | “They are not friends anymore.”  “His parents abandoned him.”  “They fell into the river.”  “She has nobody to play with.”  “The dog is ill.” |
|  | Pretend Neutral Scenarios | Not related to the above categories | “They are swimming.” |
