# Supplementary figures and images for: You Pretend, I Laugh: Associations Between Dyadic Pretend Play and Children's Display of Positive Emotions
Source: Front Psychol. 2021 Jun 23;12:669767. doi: 10.3389/fpsyg.2021.669767 (PMC8262317; doi:10.3389/fpsyg.2021.669767)

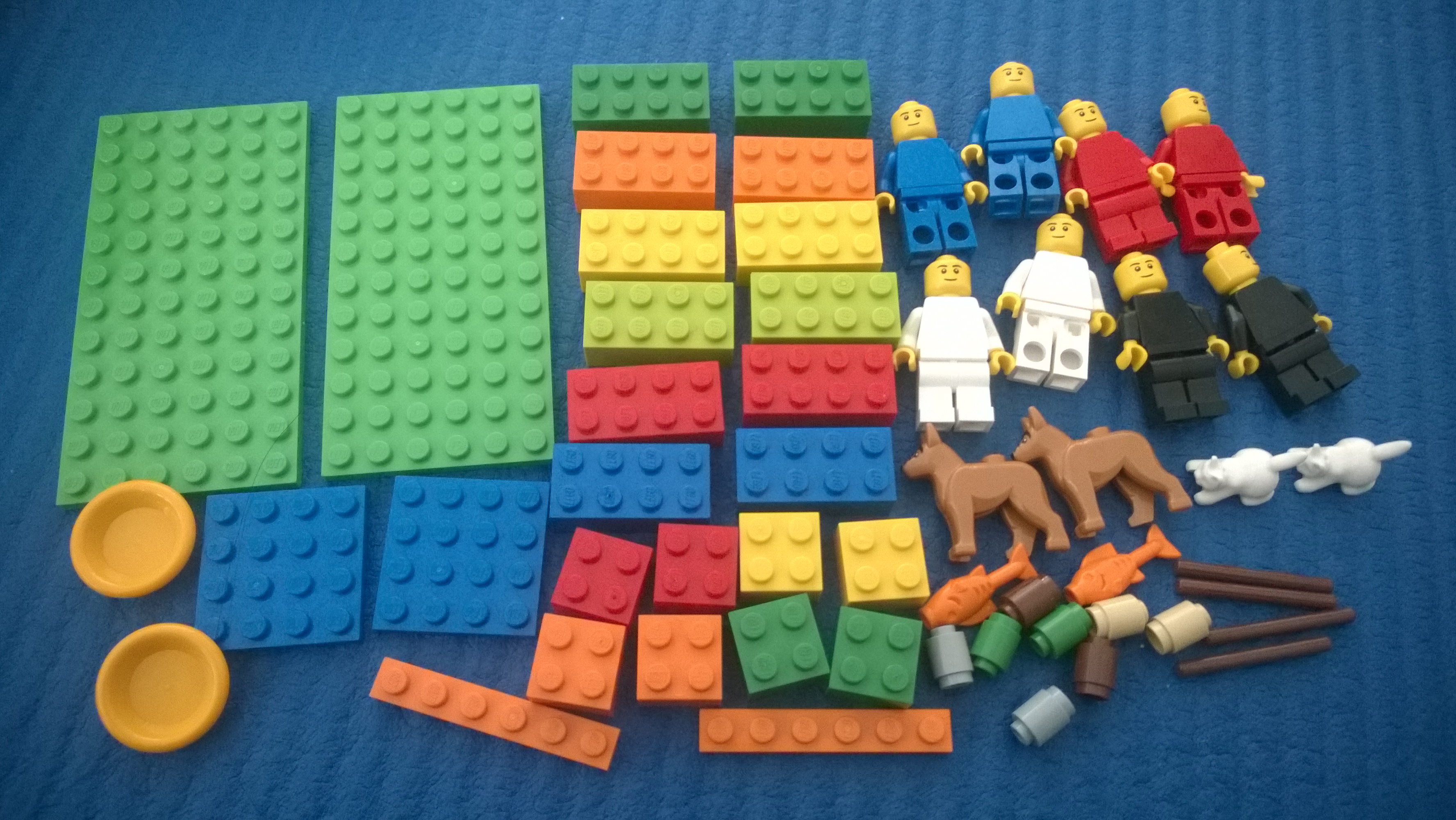

Supplement: Supplementary file 2 [file Image_1.jpeg]

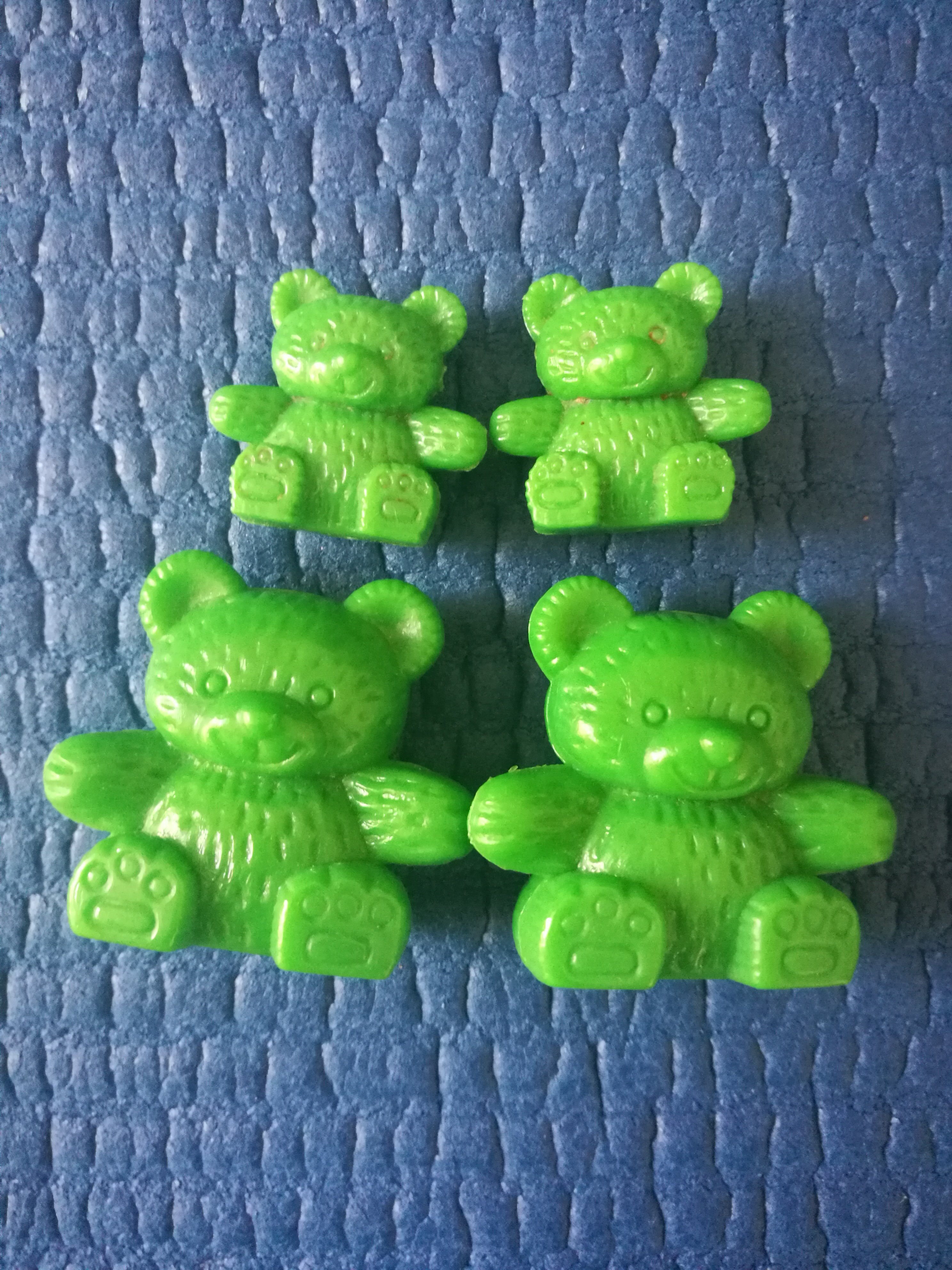

Supplement: Supplementary file 3 [file Image_2.jpeg]
